# Frequencies and subtypes of glycophorin *GYP(B-A-B)* hybrids among northern Thais, Burmese, and Karen with a previous history of malaria infection: a study in the Thailand-Myanmar border area

**DOI:** 10.7717/peerj.19589

**Published:** 2025-06-23

**Authors:** Pornsawan Srichankhot, Arissara Nakapong, Anocha Sukhanpob, Panadda Chapandoong, Amonrat Jumnainsong, Chanvit Leelayuwat, Piyapong Simtong

**Affiliations:** 1 Biomedical Science Program, Graduate School, Khon Kaen University, Khon Kaen, Thailand; 2 Centre for Research and Development of Medical Diagnostic Laboratories, Faculty of Associated Medical Sciences, Khon Kaen University, Khon Kaen, Thailand; 3 Department of Clinical Immunology and Transfusion Sciences, Faculty of Associated Medical Sciences, Khon Kaen University, Khon Kaen, Thailand

**Keywords:** *GYP(B-A-B)* hybrid alleles, Mi^a^ antigen, Blood group genotyping, Malaria

## Abstract

**Background:**

Evidence indicates that genetic variations in the *GYP(B-A-B)* hybrid genes are associated with protection against malaria. Therefore, this study aims to characterize the *GYP(B-A-B)* hybrid alleles among northern Thais, Burmese, and Karen with and without a previous history of malaria infection.

**Methods:**

A total of 709 DNA samples were genotyped to identify *GYP(B-A-B)* hybrids using PCR-sequence specific primers (PCR-SSP) combined with Sanger sequencing. Additionally, some DNA samples (*n* = 243) were also tested with high-resolution melting (HRM) analysis.

**Results:**

In our sampled populations, 14/87 (16.0%), 3/34 (8.8%), 0/16 (0%), and 1/18 (5.6%) of northern Thais, Burmese, Karen, and other minorities in Myanmar with a previous history of malaria infection, respectively, were identified with *GYP(B-A-B)* hybrid genes, whereas individuals without a history of malaria infection were 24/155 (15.5%), 5/183 (2.6%), and 4/216 (1.9%) in northern Thais, Burmese, and Karen, respectively. In the latter groups, DNA sequences showed that 17/155 (11.0%) northern Thais were GYP*Mur/GYPB heterozygotes and the other 6/155 (3.9%) were GYP*Thai/GYPB heterozygotes. The remaining one (0.6%) sample was a GYP*Mur/GYP*Mur homozygote. Among Burmese, 3/183 (1.6%) were GYP*Mur/GYPB heterozygotes and 1/183 (0.5%) was GYP*Thai/GYPB heterozygote. The remaining one (0.5%) sample being a GYP*Mur/GYP*Mur homozygote. Among Karen samples, all four were GYP*Mur/GYPB heterozygotes.

**Conclusion:**

Across all studied populations, GYP*Mur was the predominant allele, followed by GYP*Thai. In addition, genotyping results obtained by HRM were consistent with PCR-SSP combined with Sanger sequencing. A statistically non-significant association was noted for the glycophorin *GYP(B-A-B)* hybrids and malaria infection. Our findings provide insight into genetic variations of *GYP(B-A-B)* hybrid alleles among populations in the Thailand-Myanmar border area. This information could be used as a guideline to identify compatible blood products for transfusion and to prevent alloimmunization.

## Introduction

Malaria is a life-threatening disease considered to have strong selective pressure on the recent history of the human genome (Kwiatkowski, 2005). According to the World Health Organization (WHO), malaria occurs primarily in tropical and subtropical countries and is also dominant in regions of Southeast Asia, including the border areas of Thailand, especially in the forested foothills along the border with Myanmar (Konchom et al., 2003; Bharati & Ganguly, 2013). Although five species of *Plasmodium* are capable of infecting humans, in Thailand, malaria is primarily caused by *P. vivax* (47%) and *P. falciparum* (44%) (Jongruamklang et al., 2018).

The invasion of erythrocytes by merozoites is a complex and multi-step process involving receptor–ligand interactions between host cells and parasites (Prajapati & Singh, 2013; Satchwell, 2016). While *P. vivax* utilizes a single receptor, the Duffy Antigen Receptor for Chemokines (DARC), to enter red blood cells (RBCs) (Prajapati & Singh, 2013; Popovici, Roesch & Rougeron, 2020), invasion by *P. falciparum* involves multiple receptor–ligand interactions (Lopaticki et al., 2011; Jaskiewicz et al., 2019). Two main invasion pathways have been identified for *P. falciparum*: one relies on interactions with sialic-acid (SA) residues on the host cell surface and the other operates independently of these molecules (Alves-Rosa et al., 2024). The known receptors for the SA-dependent pathways are the glycophorins (GPs) which are heavily glycosylated sialoglycoproteins on RBCs (Jaskiewicz et al., 2019) that have been characterized as carrying the antigens for several human blood groups, including the MNS and Gerbich blood group systems (Alves-Rosa et al., 2024). Two important protein families on *P. falciparum* merozoites, the erythrocyte binding-like (EBL) and reticulocyte binding-like (RBL or *Pf*Rh) families, have been identified as major factors in invasion of RBCs (Lopaticki et al., 2011; Jaskiewicz et al., 2019). There are four functional EBL proteins: erythrocyte-binding antigen-175 (EBA-175), erythrocyte-binding ligand-1 (EBL-1), erythrocyte-binding antigen-140 (EBA-140), and erythrocyte-binding antigen-181 (EBA-181), capable of binding to GPs A, B, C, and D, respectively (Jaskiewicz et al., 2019). EBA-175 is the best-characterized of these and is one of the most important invasion ligands (Tolia et al., 2005; Chowdhury et al., 2018; Jaskiewicz et al., 2019). However, when GPA is inaccessible, merozoites can utilize alternative invasion pathways involving other glycophorins, such as GPB which shares structural similarities with GPA (Gaur et al., 2003; Cowman & Crabb, 2006; Jaskiewicz et al., 2019). Accordingly, recent studies have explored the role of glycophorin variants in malaria resistance. For example, rare glycophorin-deficient phenotypes such as En(a-) (lacking GPA) (Pasvol, Wainscoat & Weatherall, 1982), S-s-U- (lacking GPB) (Pasvol et al., 1982), and M^k^M^k^ (lacking GPA and GPB) (Jaskiewicz et al., 2019) significantly reduce the ability of *P. falciparum* to invade RBCs. This is because the parasites lose access to their primary receptors for invasion (Jaskiewicz et al., 2019). Similarly, GPC and GPD are also receptors for some strains of *P. falciparum*. Consequently, parasites are less efficient at invading RBCs with Ge- (lacking GPC and GPD) than invading normal RBCs (Maier et al., 2003). Another notable complex structural variant, called DUP4, creates a *GYPB-GYPA* fusion gene that alters the surface properties of RBCs and generates a hybrid glycophorin variant, called Dantu, making them less susceptible to *P. falciparum* invasion (Anstee, 2010; Leffler et al., 2017; Jaskiewicz et al., 2019) and confers a reduced risk of severe malaria (Louzada et al., 2020).

The International Society of Blood Transfusion (ISBT) Working Party for Red Cell Immunogenetics and Blood Group Terminology (ISBT WP) currently recognizes 47 blood group systems with 366 antigens on the surface of RBCs (ISBT, 2024a). The MNS blood group system (ISBT, 2024b) is the second system discovered after the ABO and is highly complex, consisting of 50 antigens. The M, N, S, and s are major antigens with 10 high-frequency and 36 low-frequency antigens (Lopez, Hyland & Flower, 2021). These antigens reside on GPA, GPB, or hybrid molecules of glycophorins. GPA with 150 amino acids is encoded by *GYPA*, which consists of seven exons. GPB with 91 amino acids is encoded by *GYPB*, which contains five exons and one pseudoexon. Together with a third gene in this family, *GYPE,* these genes form a 350-kb gene cluster on chromosome 4q31.21 (Lopez, Hyland & Flower, 2021; ISBT). *GYPE* has four exons plus two pseudoexons. Although this gene may encode GPE, this antigen may not be expressed on the RBC surface. These genes are highly homologous and have a similar exon-intron organization (Wei et al., 2018; Lopez, Hyland & Flower, 2021). This leads them to easily generate hybrid glycophorin variant alleles through two main mechanisms, unequal crossing-over and gene conversion between the *GYPA*, *GYPB,* and occasionally, *GYPE* genes (Jongruamklang et al., 2020; Lopez, Hyland & Flower, 2021). MNS hybrid glycophorins include *GYP(A-B*), *GYP(B-A), GYP(A-B-A*), and *GYP(B-A-B)* (Wei et al., 2018). The common variants found in Southeast Asia are the *GYP(B-A-B*) alleles (Jongruamklang et al., 2020; Nathalang et al., 2024). While exon 3 of *GYPA* is fully functional and expressed, exon 3 of *GYPB* is a pseudoexon, which is not expressed because of a point mutation in the splice acceptor site (Heathcote, Carroll & Flower, 2011). In the case of *GYP(B-A-B),* hybrid variants arise from gene conversion events between *GYPA* and *GYPB* genes (Reid, 1994; Wei et al., 2018). These lead to the insertion of a homologous segment from the exon 3 region and the 5′ end of intron 3 of the *GYPA* gene into the *GYPB* gene. This area is a hotspot for gene conversion events (Wei et al., 2018). During these gene conversion events, alterations in consensus splice sequences may occur and facilitate transcription of the *GYPB* pseudoexon 3. Hence, it allows the translation of a sequence of amino acids that would normally be spliced out (Reid, 1994). Each of the *GYP(B-A-B)* hybrid alleles, including GYP*HF, GYP*Mur, GYP*Bun, GYP*Hop, and GYP*Kip (Lomas-Francis, 2011; Lin et al., 2019), differs from each other by the location and the length of *GYPA* nucleotide insertions (Wei et al., 2016a). Additionally, a novel GYP*Bun-like allele (designated as GYP*Thai), with a shorter insertion of *GYPA*, is another of the *GYP(B-A-B)* hybrid variants (Wei et al., 2016b; Jongruamklang et al., 2020). Therefore, each *GYP(B-A-B)* hybrid allele expresses a set of several low-incidence antigen profiles (Lomas-Francis, 2011; Wei et al., 2016a; Lopez, Hyland & Flower, 2021). To date, over 30 hybrid genes are known in the MNS blood group system (Lopez, Hyland & Flower, 2021). The GP(B-A-B) hybrid glycophorins are GP.HF, GP.Mur, GP.Bun, GP.Hop, and GP.Kip (Wei et al., 2018). Among them, GP.Mur is the most common in Southeast Asia (Jongruamklang et al., 2018; Nathalang et al., 2024). The presence of this variant leads to the upregulation of band 3 on the surface of RBCs, making them more resistant to osmotic stress. Thus, it may provide resistance to *P. falciparum* (Hsu et al., 2009, p. 3; (Lomas-Francis, 2011; Heathcote, Carroll & Flower, 2011). Alloantibodies to hybrid glycophorin antigens are far more frequently implicated in immediate and delayed hemolytic transfusion reactions (HTRs) and hemolytic disease of the fetus and newborn (HDFN) in Southeast Asian than Caucasian and African populations (Poole & Daniels, 2007; Heathcote, Carroll & Flower, 2011; Makroo et al., 2016). All glycophorin *GYP(B-A-B)* hybrids along with GPs from two other variants, GP.MOT encoded by *GYP(B-A)* and GP.Vw and GP.Hut encoded by *GYP(A-B-A)*, express the Mi^a^ antigen (MNS7) (Nathalang et al., 2024). This antigen is found in 4.7–22.3% of Thais depending on the region (Makroo et al., 2016; Intharanut et al., 2017; Jongruamklang et al., 2018; Romphruk et al., 2019; Khosidworachet et al., 2022). Within Southeast Asia populations, the frequencies range from 1.75−9.6% (Musa et al., 2012; Hsu et al., 2013). In East Asia, frequencies are 4.19% among Taiwanese (Hsu et al., 2013) and 6.5% among Southern Han Chinese (Wei et al., 2018), with a higher frequency reported in Chinese from Guangzhou at 9.7% (Wei et al., 2016b). In contrast, the antigen is seen in 0.1% of Indians (Makroo et al., 2016), 0.22% of Australians (Lopez et al., 2020), and <0.1% of Caucasian, African, and Japanese populations (Mohn, Lambert & Rosamilia, 1961; Mohn et al., 1963; Makroo et al., 2016).

The border area of Thailand-Myanmar is region where malaria is endemic. Glycophorin variants can affect susceptibility to malaria, but no studies have been conducted to determine the distribution of hybrid glycophorin polymorphisms in this region. In particular, glycophorin *GYP(B-A-B)* hybrids are prevalent variants within the MNS blood group system in Southeast Asia. For these reasons, molecular-based techniques, including PCR-sequence specific primer (PCR-SSP) combined with Sanger sequencing and high-resolution melting (HRM) analysis were performed to distinguish the *GYP(B-A-B)* hybrid genotypes. The aim was to investigate the distribution of such genotypes in the populations along the Thailand-Myanmar border and its association with previous history of malaria infection. The results obtained in this study will provide insight into the association between glycophorin *GYP(B-A-B)* hybrids and malaria infection. Understanding the distribution and function of these variants may support the recognition of at-risk individuals and may inform targeted malaria control interventions. Potentially, this information may enhance malaria treatment and control strategies. Additionally, molecular techniques that have been applied in this study could be adopted for molecular blood group screening to provide genotyped-matched donor blood units to prevent alloimmunization, particularly among chronically transfused patients.

## Materials & Methods

### Samples

**Figure 1:**
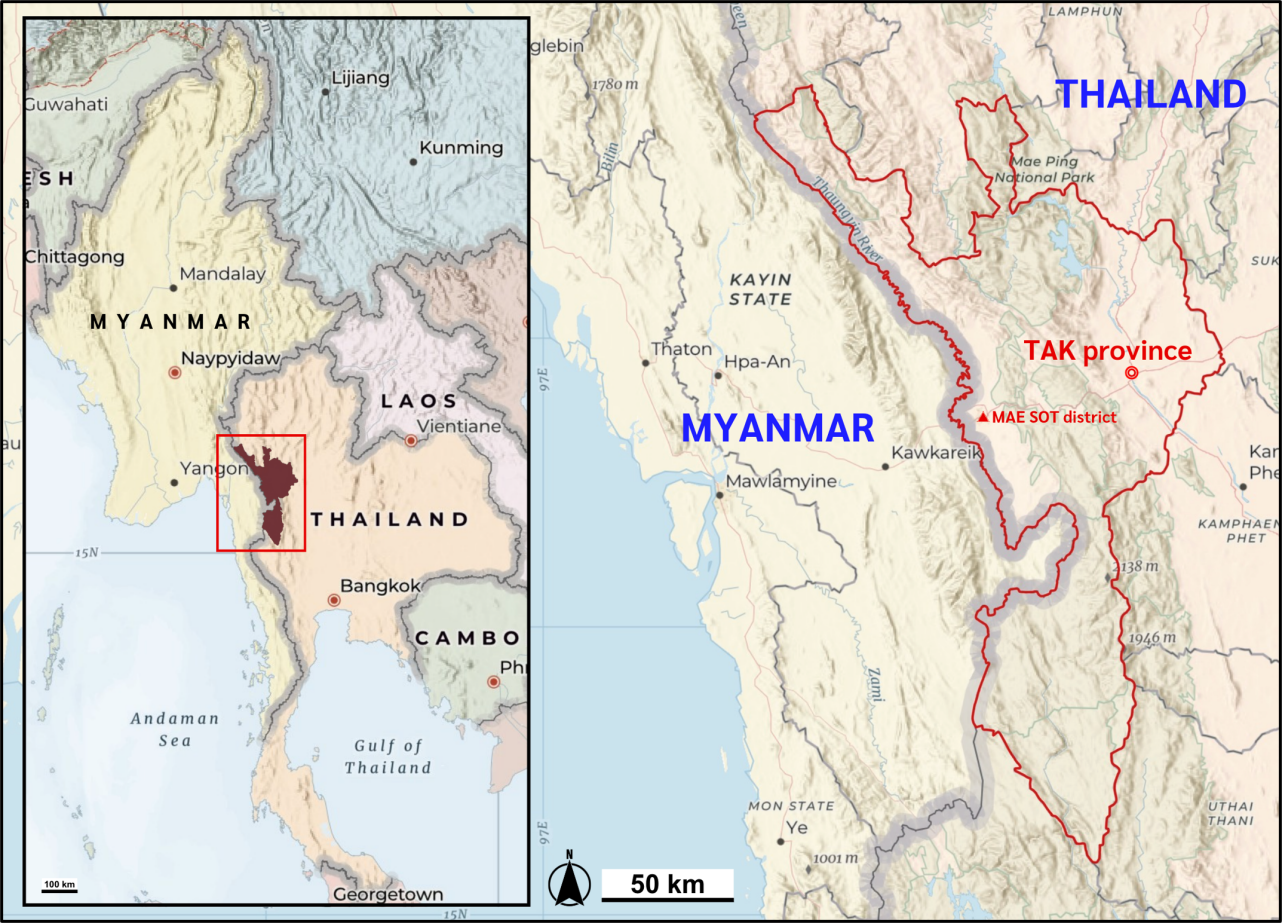
Map of the study location. Mae Sot district, Tak Province of Thailand shares the international border with the Kayin state of Myanmar. Map data ©2025 Esri, USGS — NOSTRA, Esri, TomTom, Garmin, FAO, NOAA, USGS. Created using ArcGIS Online Map Viewer (https://www.arcgis.com/apps/mapviewer/index.html).

This study used a total of 709 DNA specimens, left over from a previous project (Duangchanchot et al., 2009), from individuals in the Thailand-Myanmar border region (Fig. 1). The samples came from 87 northern Thais, 34 Burmese, 16 Karen, and 18 from other minorities in Myanmar: all individuals had a previously recorded history of malaria infection. As controls, we used samples from 155 northern Thais, 183 Burmese, and 216 Karen individuals, all without a previous history of malaria infection. All consenting individuals were interviewed regarding their history of malaria infection and the ethnic origin of their parents and grandparents between 25 July 2006 and 12 June 2007. All leftover DNA samples in this study were accessed on 1 April 2024. Ethical approval of the study protocol was obtained from the Institutional Review Board (IRB) of Khon Kaen University, Thailand (HE672068).

Our genomic DNA samples were consistently stored at −20 °C in Tris-EDTA buffer until analysis. DNA concentration and purity were quantified utilizing a NanoDrop™ 2000c Spectrophotometer (Thermo Fisher Scientific, Waltham, MA, USA). Additionally, the quality of each DNA sample was evaluated through agarose-gel electrophoresis within the PCR-SSP analysis. To ensure the validity of negative results, internal control primers were incorporated into each PCR reaction. Samples displaying significant degradation, indicated by the absence of internal control bands, were excluded from the study.

### Molecular screening for glycophorin *GYP(B-A-B)* hybrids using PCR-SSP

The glycophorin *GYP(B-A-B)* hybrids (GYP*HF, GYP*Mur, GYP*Bun, GYP*Thai, GYP*Hop, and GYP*Kip) were identified using an in-house PCR-SSP. The specific pair of primers: forward primer, 5′-GCGGTCCCTTTCTCAACTTCTCTTATATCCAGATAA-3′ and reverse primer, 5′-GCGGTGAGCAACTATTTAAAACTAAGAACATACCGG-3′ similar to those in a previous study (Lin et al., 2019), amplified a 158 bp PCR product of GYP*HF, GYP*Mur, GYP*Bun, GYP*Hop, GYP*Kip, and GYP*Hut. A 434-bp region of the human growth hormone gene was co-amplified as an internal control using the following primers: forward, 5′-TGCCTTCCCAACCATTCCCTTA-3′ and reverse, 5′-CCACTCACGGATTTCTGTTGTGTTTC-3′ (Palacajornsuk et al., 2007).

**Figure 2:**
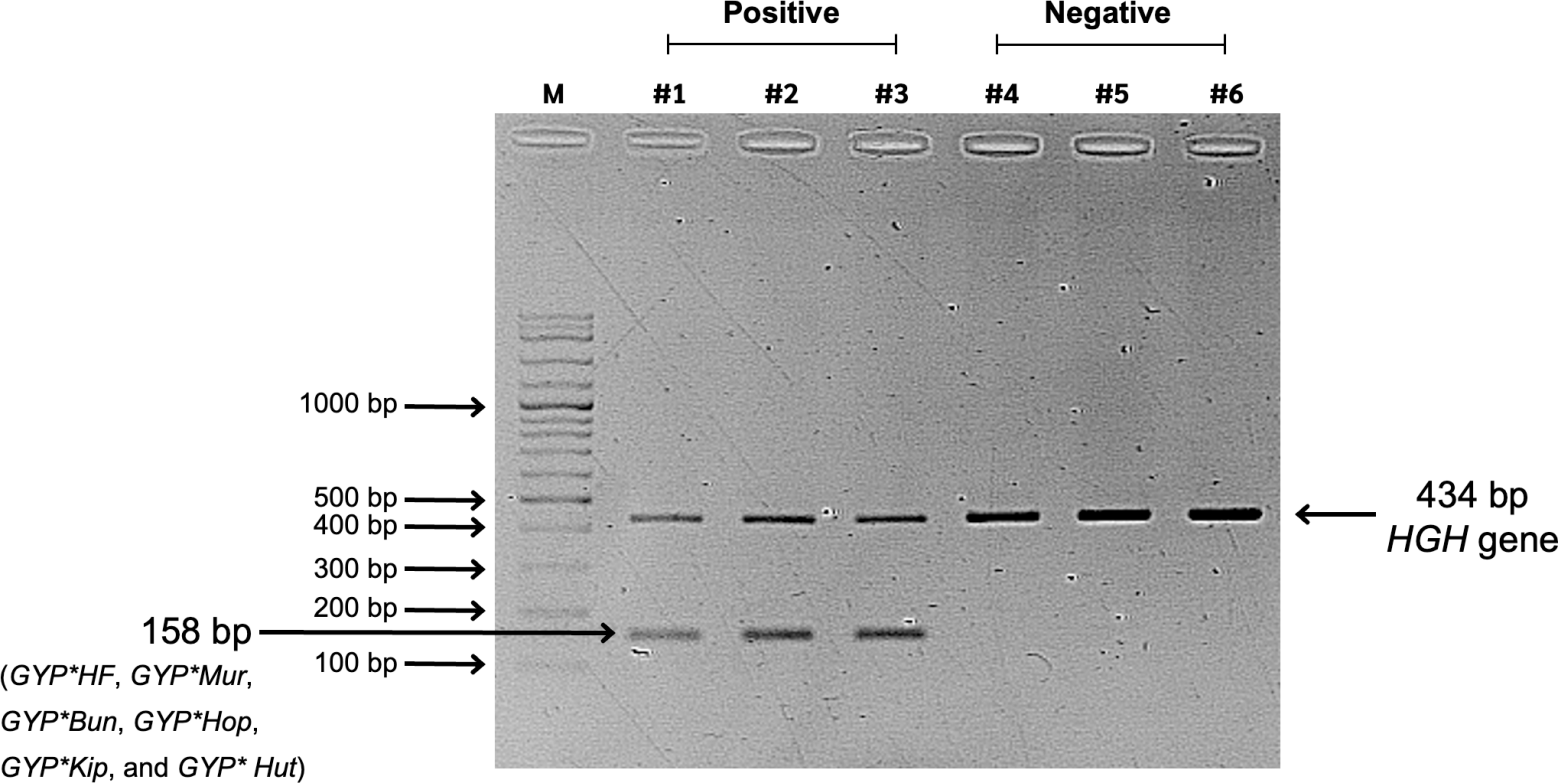
Screening results of glycophorin *GYP(B-A-B)* hybrids using PCR-SSP. Lane M represents a 100-bp DNA molecular weight ladder (VC 100 bp Plus DNA Ladder, NL1407, Vivantis). Lanes #1, #2, and #3 show positive *GYP(B-A-B)* hybrid samples amplified using two primer sets: specific primers (GYP*HF, GYP*Mur, GYP*Bun, GYP*Hop, GYP*Kip, and GYP*Hut) and internal control primers (human growth hormone, *HGH*, gene), resulting in PCR bands of 158 bp and 434 bp, respectively. Lanes #4, #5, and #6 represent negative *GYP(B-A-B)* hybrid samples, showing only the internal control band with 434 bp.

The PCR reaction mixes with final volume of 13 µl contained 100 ng of DNA, 0.5 µM of the specific primers, 0.1 µM of the internal control primers, and 1x Master Mix PCR (67 mM Tris-HCL pH 8.8, 17 mM ammonium sulfate, 0.1% Tween 20, 0.2 mM dNTPs (Vivantis, Kuala Lumpur, Malaysia), and 2 mM MgCl_2_). The PCR was then performed using a thermocycler (Applied Biosystems Veriti™, Thermal Cycler, Life Technologies, Foster City, CA, USA) with following conditions: initial denaturation for 2 min at 96 °C followed by 5 cycles of 30 s at 96 °C, 60 s at 65 ° C, and 40 s at 72 °C; then 21 cycles of 30 s at 96 °C, 60 s at 60 °C, and 40 s at 72 °C; then 4 cycles of 30 s at 96 °C, 1.15 min at 55 °C, and 2 min at 72 ° C; followed by a final extension for 10 min at 72 ° C. After the amplification, the PCR products were analyzed by visualizing the PCR bands on 1.5% agarose gels under UV light using a gel documentation system (Syngene, Maryland, DE, USA) (Fig. 2).

### Characterizing *GYP(B-A-B)* hybrid alleles using Sanger sequencing

**Figure 3:**
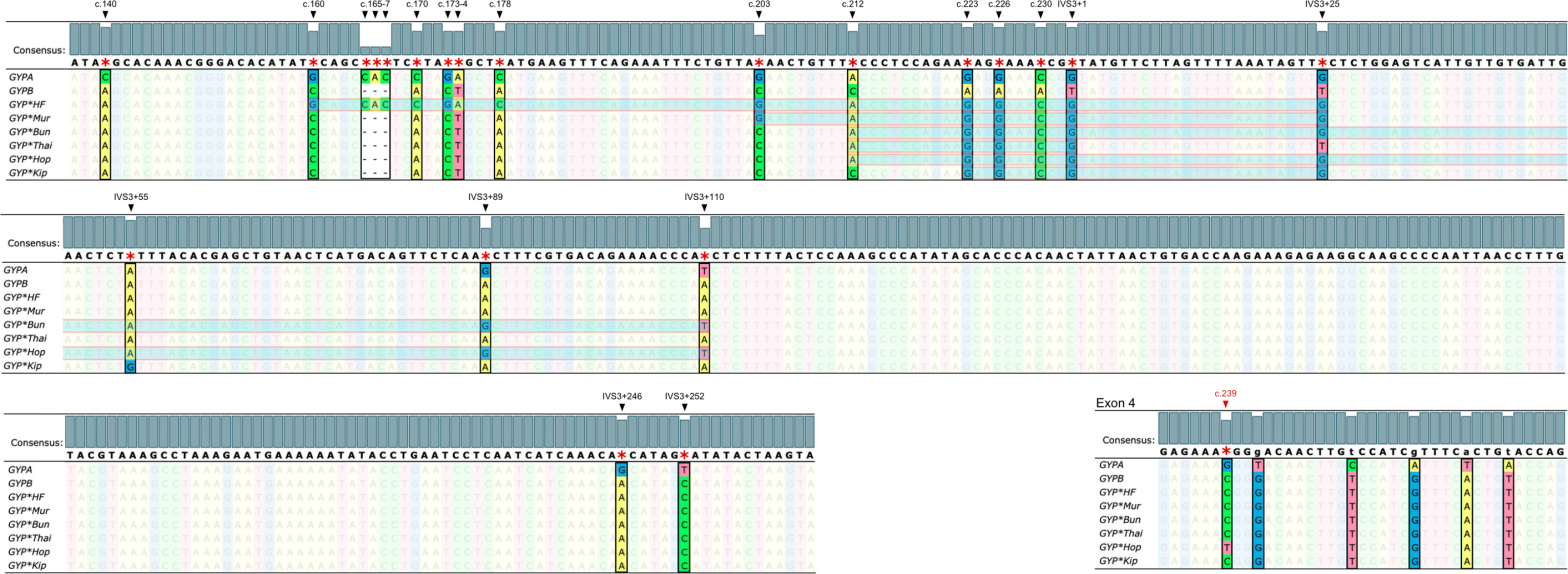
Nucleotide sequence alignment of *GYPA*, *GYPB*, and *GYP(B-A-B)*. The nucleotide sequences of exon 3, part of intron 3, and exon 4 of *GYPA*, *GYPB*, and *GYP(B-A-B)* were retrieved from GenBank and were aligned using Unipro UGENE: a unified bioinformatics toolkit. The figure displays the consensus sequences and the polymorphic positions at c.140, c.160, c.165, c.166, c.167, c.170, c.173, c.174, c.178, c.203, c.212, c.223, c.226, c.230, IVS3+1, IVS3+25, IVS3+55, IVS3+89, IVS3+110, IVS3+246, IVS3+252, and c.239. Each *GYP(B-A-B)* subtype has distinct *GYPA* nucleotide insertions (indicated by highlighted parts). GYP*HF shows a 98 bp insertion from c.160 to IVS3+25. GYP*Mur has a 55 bp insertion from c.203 to IVS3+25. GYP*Bun and GYP*Hop share an identical 131 bp insertion from c.212 to IVS3+110. To distinguish GYP*Bun from GYP*Hop, a single nucleotide polymorphism (SNP) located in exon 4 needed to be observed (c.239C = GYP*Bun, c.239T = GYP*Hop). GYP*Thai has a 22 bp insertion from c.212 to IVS3+1. Whereas GYP*Kip shows a 35 bp insertion from c.223 to IVS3+25.

**Figure 4:**
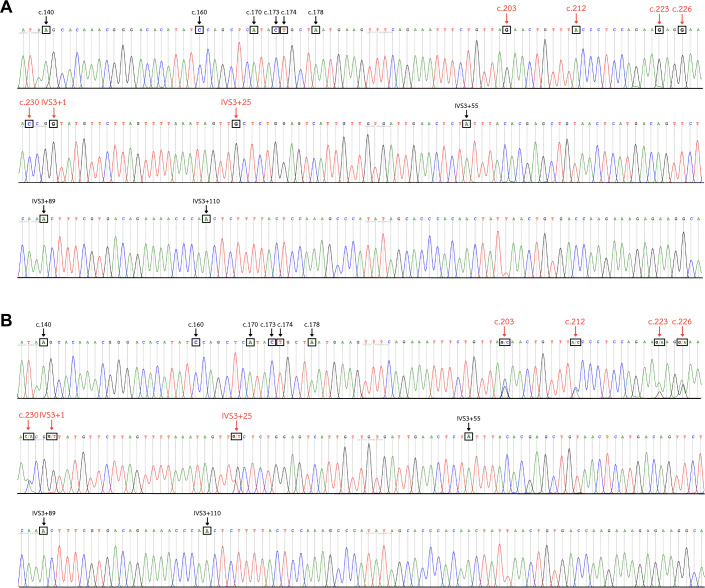
DNA sequence chromatogram of exon 3 and part of intron 3 regions of *GYP(B-A-B)* hybrids. (A) The GYP*Mur/ GYP*Mur homozygote sample displays single unambiguous peaks of the chromatogram of the *GYPA* sequence at c.203, c.212, c.223, c.226, c.230, IVS3+1, and IVS3+25 (red arrow). (B) The GYP*Mur/GYPB heterozygote sample consists of 2 alleles, GYP*Mur and *GYPB*, which display double peaks at c.203, c.212, c.223, c.226, c.230, IVS3+1, and IVS3+25 (red arrow).

The identification of *GYP(B-A-B)* hybrid alleles was performed using DNA sequencing analysis. Pseudoexon 3 of *GYPB*, exon 3 of *GYP(B-A-B)*, and parts of the adjacent intron regions were amplified to yield a 383-bp product using a forward primer, 5′-CTGGGAGGGATGTGGGAGAA-3′ and reverse primer, 5′-ACAAAGGTTAATTGGGGCTTGC-3′ (Haer-Wigman et al., 2013). The PCR conditions were as mentioned above. Sanger sequencing was then conducted to determine the DNA sequence using fluorescent dye-terminator sequencing on an analyzer (ABI Prism™ 3730XL DNA sequencers; Bio Basic Inc., Markham, ON, Canada). The nucleotide differences between *GYPA*, *GYPB*, and *GYP(B-A-B)* hybrid alleles were compared against reference sequences in the GenBank database: *GYPA* (NG_007470.3), *GYPB* (M60708.1), GYP*HF (M81079.1), GYP*Mur (AF090739.1), GYP*Bun (M60710.1), GYP*Thai (KR363627.1), GYP*Hop (KR815995.1), and GYP*Kip (KF501485.2) (Fig. 3). The chromatograms of DNA sequences were analyzed with Unipro UGENE: a unified bioinformatics toolkit (Okonechnikov et al., 2012) (Fig. 4).

### HRM genotyping assay

The HRM assay was performed to distinguish *GYP(B-A-B)* hybrids using the distinct length and GC percentage of each hybrid. The specific primers: forward, 5′-ACGCAGTCACCTCATTCTTGTT-3′ and reverse, 5′-GGCTTTGGAGTAAAAGAGTTG GG-3′ (Wei et al., 2018) were used to amplify the pseudoexon 3 of *GYPB*, exon 3 of *GYP(B-A-B)*, and parts of the adjacent intron regions. The resulting PCR products were 270 bp long for *GYPB*, GYP*Mur, and GYP*Bun and 273 bp for GYP*HF. A control panel, characterized by DNA sequencing was used to assess the assay, including wild-type *GYPB/GYPB* (*n* = 4), GYP*Mur/GYP*Mur homozygotes (*n* = 3), GYP*Mur/*GYPB* heterozygotes (*n* = 6), and GYP*Thai/*GYPB* heterozygotes (*n* = 6). The PCR reaction had a final volume of 25 uL containing 10 uL of 2X Precision Melt Supermix (Bio-Rad, Hercules, CA, USA), 2.5 uL of DNA (10 ng/uL), 0.5 uL of each 5 uM forward and reverse primers, and 11.5 uL of ultra-pure water (Bio Basic Inc., Toronto, Canada). The PCR step was performed next using a real-time PCR cycler (CFX96™ Real-Time system C1000Touch™ Thermal cycler; Bio-Rad) under the following conditions: initial activation at 95 °C for 5 min followed by 40 cycles of denaturation at 95 °C for 10 s and annealing/extension at 65 °C for 30 s. The HRM cycle was then started by gradually increasing the temperature by 0.1 °C every 2 s from 73 °C to 83 °C.

**Figure 5:**
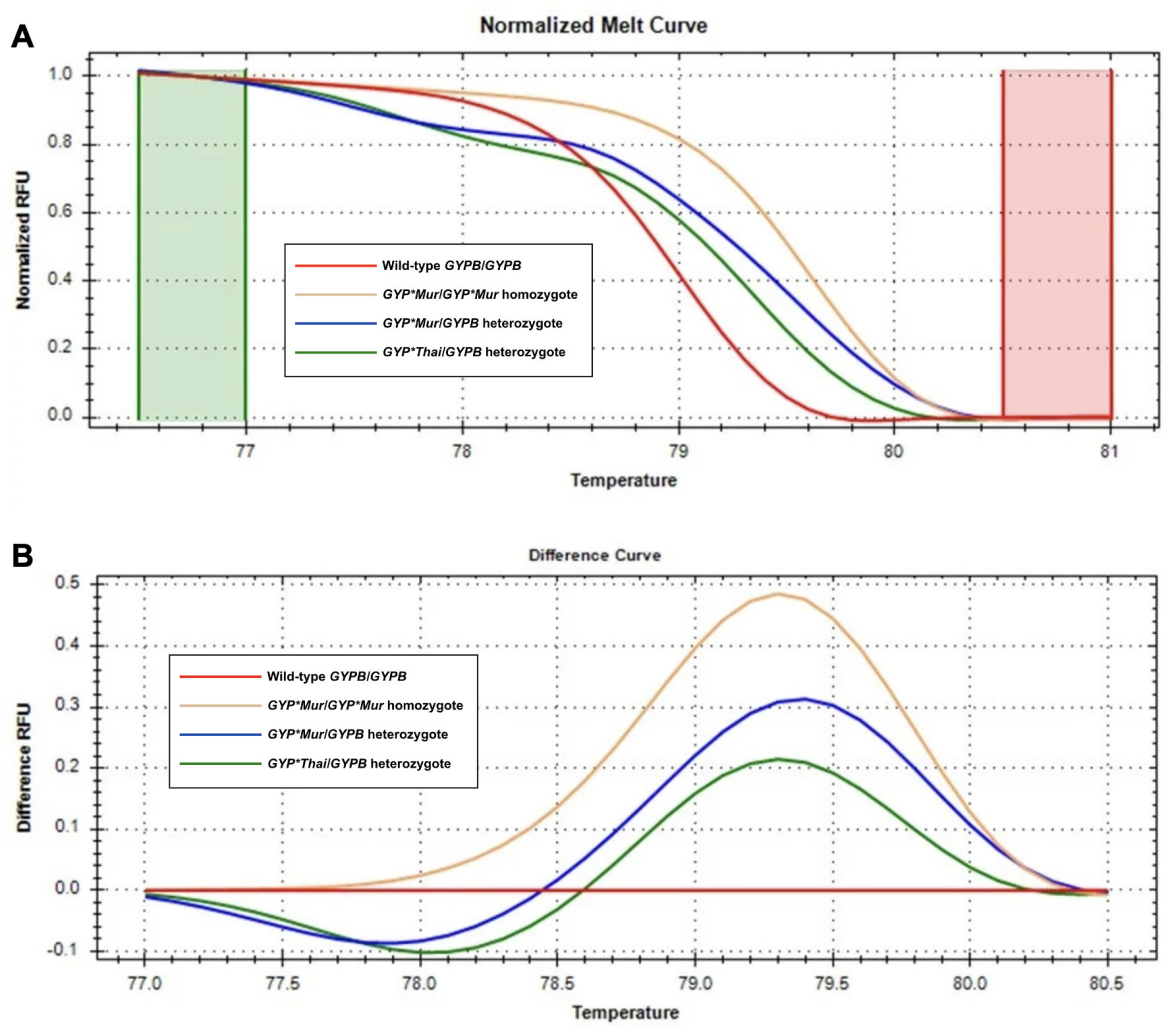
Melting curve profiles of wild-type *GYPB/GYPB* and *GYP(B-A-B)* hybrids by HRM analysis. Fluorescence intensity for all samples was plotted in relative fluorescence units (RFU). (A) The normalized melt curve was adjusted to relative values of 1.0 and 0 to remove the background fluorescence and enhance the visibility of subtle melt profile differences. (B) The difference curve was plotted to visually amplify differences between melt profiles of different subtypes. Four different subtypes ((*GYPB/GYPB* (red), GYP*Mur/GYP*Mur (yellow), GYP*Mur/GYPB (blue), and GYP*Thai/GYPB (green)) were distinguished regarding both melting temperature shifts and curve shapes. Homozygous variants are characterized primarily by the temperature shift, while heterozygotes are commonly identified by a change in melt curve shape due to base-pairing mismatches.

After the melting temperature (Tm) was reached, double-stranded DNA was denatured and EvaGreen was released, causing a dramatic decrease in fluorescence intensity. The fluorescence change rate was monitored and analyzed using Bio-Rad Precision Melt Analysis software (Bio-Rad Laboratories Inc.). The melting curve profiles of unknown samples were compared to the DNA control samples. Data that were similar to each other were clustered and color-coded by the software for easy visualization (Fig. 5). A confidence threshold was set at 90%. Samples showing a percent confidence below 90 were labeled as inconclusive (Lopez et al., 2015; Wei et al., 2018; Nathalang, Intharanut & Chidtrakoon, 2021). Therefore, further investigation by direct DNA sequencing was required.

### Statistical analysis

The frequencies of genotypes, alleles, and predicted Mi^a^ antigen were calculated and expressed in numbers, percentages, and 95% confidence intervals (CI). A chi-square test was performed to compare the frequencies of genotypes, alleles, and predicted Mi^a^ antigen among different populations and the distribution of glycophorin *GYP(B-A-B)* hybrids between the individuals with and without a previous history of malaria infection. Odds ratios (OR) and 95% CI were also computed. Any *p*-value less than 0.05 was considered statistically significant. All statistical analyses were conducted using IBM SPSS Statistics version 28.0.1.1. (IBM Corporation, Armonk, NY, USA)

## Results

### Frequency of glycophorin *GYP(B-A-B)* hybrids using PCR-SSP

A total of 709 DNA samples were tested for glycophorin *GYP(B-A-B)* hybrids using PCR-SSP. Among individuals with a previous history of malaria infection, frequencies of such hybrids were 14/87 (16.0%), 3/34 (8.8%), 0/16 (0%), and 1/18 (5.6%) among northern Thais, Burmese, Karen, and other minorities in Myanmar, respectively. Meanwhile, frequencies of hybrid glycophorin genes among individuals without a history of malaria infection were 24/155 (15.5%), 5/183 (2.6%), and 4/216 (1.9%) in northern Thais, Burmese, and Karen, respectively. All detected glycophorin hybrids were Mi^a^-bearing. Therefore, these individuals are also considered Mi^a^ antigen positive. Burmese and Karen populations presented significantly lower frequencies than those of northern Thais. Although no significant difference was found between them (Table 1).

**Table 1:** Summary of genotype and allele frequencies of glycophorin *GYP(B-A-B)* hybrids among individuals with and without a previous history of malaria infection.

Populations	Genotypes						Predicted Mi^a^		Alleles					
	GYP*Mur/GYP*Mur		GYP*Mur/GYPB		GYP*Thai/GYPB				***GYPB***		GYP*Mur		GYP*Thai	
	**Number of samples**	**Population frequency,% (95% CI)**	**Number of samples**	**Population frequency,% (95% CI)**	**Number of samples**	**Population frequency,% (95% CI)**	**Number of samples**	**Population frequency,% (95% CI)**	**Number of samples**	**Population frequency,% (95% CI)**	**Number of samples**	**Population frequency,% (95% CI)**	**Number of samples**	**Population frequency,% (95% CI)**
With a previous history of malaria infection														
Northern Thais (*n* = 87)	1	1.1 (0.0–6.2)	12	13.8 (7.3–22.9)	1	1.1 (0.0–6.2)	14	16.0 (9.1–25.5)	159	91.4 (86.2–95.1)	14	8.0 (4.5–13.1)	1	0.6 (0.0-3.2)
Burmese (*n* = 34)	0	0 (0.0–10.3)	2	5.9 (0.7–19.7)	1	2.9 (0.1–15.3)	3	8.8 (1.9–23.7)	65	95.6 (87.6–99.1)	2	2.9 (0.4–10.2)	1	1.5 (0.0–7.9)
Karen (*n* = 16)	0	0 (0.0–20.6)	0	0 (0.0–20.6)	0	0 (0.0–20.6)	0	0 (0.0–20.6)	32	100 (89.1–100.0)	0	0 (0.0–10.9)	0	0 (0.0–10.9)
Minorities in Myanmar^#^ (*n* = 18)	0	0 (0.0–18.5)	1	5.6 (0.1–27.3)	0	0 (0.0–18.5)	1	5.6 (0.1–27.3)	35	97.2 (85.5–99.9)	1	2.8 (0.1–14.5)	0	0 (0.0–9.7)
Without a previous history of malaria infection														
Northern Thais (*n* = 155)	1	0.6 (0.0–3.5)	17	11.0 (6.5–17.0)	6	3.9 (1.4–8.2)	24	15.5 (10.2–22.2)	285	92.0 (88.3–94.7)	19	6.1 (3.7–9.4)	6	1.9 (0.7–4.2)
Burmese (*n* = 183)	1	0.5 (0.0–3.0)	3	1.6 ^d^ (0.3–4.7)	1	0.5^a^ (0.0–3.0)	5	2.6^d^ (0.9–6.3)	360	98.3^d^ (96.5–99.4)	5	1.4^d^ (0.4–3.2)	1	0.3^b^ (0.0–1.5)
Karen (*n* = 216)	0	0 (0.0–1.7)	4	1.9^d^ (0.5–4.7)	0	0^c^ (0.0–1.7)	4	1.9^d^ (0.5–4.7)	428	99.1^d^ (97.6–99.7)	4	0.9^d^ (0.3–2.4)	0	0^c^ (0.0–0.9)

**Notes.**

a Significant difference from northern Thais at *p*-value = 0.032.

b Significant difference from northern Thais at *p*-value = 0.033.

c Significant difference from northern Thais at *p*-value = 0.004.

d Significant difference from northern Thais at *p*-value < 0.001.

# The minority ethnic groups in Myanmar, such as Kachin, Shan, Hmong, Mon, and Rakhine.

### Genotype and allele frequencies of glycophorin *GYP(B-A-B)* hybrids using Sanger sequencing

All 51 positive glycophorin *GYP(B-A-B)* hybrid samples were further analyzed using Sanger sequencing and polymorphic positions in each subtype noted (Fig. 3). The DNA sequencing analysis results (Fig. 4) among individuals with a previous history of malaria infection, genotypes detected were as follows: 1/87 (1.1%) GYP*Mur/GYP*Mur homozygote, 12/87 (13.8%) GYP*Mur/GYPB heterozygotes, and 1/87 (1.1%) of GYP*Thai/GYPB heterozygote in northern Thais. For Burmese 2/34 (5.9%) were GYP*Mur/GYPB heterozygotes and 1/34 (2.9%) was a GYP*Thai/GYPB heterozygote. No glycophorin *GYP(B-A-B)* hybrids were detected in the Karen. Among other minorities in Myanmar, only 1/18 (5.6%) was a GYP*Mur/GYPB heterozygote. In individuals without a previous history of malaria infection, genotype frequencies were as follows: northern Thais 1/155 (0.6%) GYP*Mur/ GYP*Mur homozygote, 17/155 (11.0%) GYP*Mur/GYPB heterozygotes, and 6/155 (3.9%) GYP*Thai/GYPB heterozygotes. For Burmese 1/183 (0.5%) was a GYP*Mur/ GYP*Mur homozygote, 3/183 (1.6%) were GYP*Mur/GYPB heterozygotes and 1/183 (0.5%) was GYP*Thai/GYPB heterozygote. Of the Karen samples 4/216 (1.9%) were GYP*Mur/GYPB heterozygotes. The genotype frequencies of GYP*Mur/GYPB and GYP*Thai/GYPB within Burmese and Karen populations were significantly different from those of northern Thais. Therefore, the frequencies of GYPB, GYP*Mur, and GYP*Thai alleles in Burmese and Karen populations are also significantly different from northern Thais. In this study, across all studied populations, both with and without a previous history of malaria infection, GYP*Mur was the most common allele, followed by GYP*Thai (Table 1).

### Differentiation of glycophorin *GYP(B-A-B)* hybrid genotypes using HRM assay

The HRM assay was conducted in some of the DNA samples (*n* = 243) to observe the concordance with PCR-SSP and Sanger sequencing. The DNA samples were genotyped using the HRM assay to distinguish among glycophorin *GYP(B-A-B)* hybrids based on their characteristics, including the PCR-product length and GC percentage. The results were completely in concordance with those obtained from PCR-SSP combined with Sanger sequencing.

### Association between glycophorin *GYP(B-A-B)* hybrids and malaria infection

The association between glycophorin *GYP(B-A-B)* hybrids and malaria infection was investigated by comparing the distribution of these hybrids in individuals of three ethnicities with and without a previous history of malaria infection. The results indicated no statistically significant differences in the distribution of glycophorin *GYP(B-A-B)* hybrids between all three ethnicities among Northern Thais, Burmese, and Karen (Table 2).

**Table 2:** Comparison of glycophorin *GYP(B-A-B)* hybrids in individuals of three ethnicities with and without a previous history of malaria infection.

Population	With a previous history of malaria infection			Without a previous history of malaria infection			*p*-value	OR	95% CI
	**Number of samples**	***GYP(B-A-B)*** **positive**	**Population frequency (%)**	**Number of samples**	***GYP(B-A-B)*** **positive**	**Population frequency (%)**			
Northern Thais	87	14	16.0	155	24	15.5	0.901	1.047	0.510–2.148
Burmese	34	3	8.8	183	5	2.6	0.083	3.445	0.783–15.154
Karen	16	0	0	216	4	1.9	0.583	NT	NT

**Notes.**

NT, no statistics were computed due to the number of zero values.

## Discussion

This study documented the distribution of glycophorin *GYP(B-A-B)* hybrids in populations with and without a previous history of malaria infection in an area along the border between Thailand and Myanmar, an endemic malaria region. Ethnic groups evaluated included northern Thais, Burmese, and Karen. Published evidence suggests that certain glycophorin variants within the MNS blood group system are protective factors against the invasion of RBCs by *P. falciparum* merozoites due to the absence or structural variation of their primary RBC receptors. Notable examples include Ena- RBCs (lack of GPA) (Pasvol, Wainscoat & Weatherall, 1982), S-s-U- RBCs (lack of GPB) (Pasvol et al., 1982), M^k^M^k^ RBCs (lack of GPA and GPB) (Jaskiewicz et al., 2019), which exhibit varying degrees of resistance because these receptors are essential for merozoite ligands. Additionally, hybrid glycophorin variants affect parasite binding by modifying RBC surface properties. For example, the Dantu protein (GP(A-B) hybrid) reduces severe malaria risk by 43% among heterozygotes and 74% among homozygotes *via* alterations in RBC tension and receptor availability (Anstee, 2010; Leffler et al., 2017; Jaskiewicz et al., 2019), while GP.Mur protein (GP(B-A-B) hybrid) has been associated with enhanced band 3 expression, potentially influencing efficiency of merozoite invasion (Hsu et al., 2009, p. 3; Lomas-Francis, 2011; Heathcote, Carroll & Flower, 2011). Our finding revealed no statistically significant association between the presence of glycophorin *GYP(B-A-B)* hybrids and malaria infection in the studied populations along the Thailand-Myanmar border. This suggests that there could be specific glycophorin variants that can affect malaria susceptibility. Not all variants may offer the same level of protection, as GPB-negative cells exhibited moderate resistance to malaria invasion (Gaur et al., 2003) but demonstrated almost complete resistance to GPA-negative cells (Salinas, Paing & Tolia, 2014).

However, the lack of statistically significant association in this study may be due to the limited sample size or other confounding factors that could not be controlled such as specific *Plasmodium* species. We were unable to identify the specific species of *Plasmodium* in this study. According to the malaria infection history of all consenting individuals was self-reported through the interview. We were also unable to accurately assess individual malaria exposure levels, which may have influenced the study’s outcomes. Further studies with larger sample sizes and species-specific analysis are necessary to explore this relationship comprehensively.

Even though glycophorin *GYP(B-A-B)* hybrids are common variants found in Southeast Asia. This is the first study to identify such hybrids, Mi^a^-bearing hybrids, in northern Thais (residing in Tak province), Burmese, and Karen people using molecular techniques. Such hybrids were presented in 15.5%, 2.6%, and 1.9% of northern Thais, Burmese, and Karen, respectively. The frequency of *GYP(B-A-B)* hybrids in northern Thais is therefore significantly higher than in Burmese and Karen people. These frequencies could also be considered as predicted Mi^a^ frequencies of each population because all hybrids assessed were Mi^a^-bearing (Nathalang et al., 2024). Accordingly, alloantibodies against these glycophorin hybrids and Mi^a^ may also be higher in northern Thais than in Burmese and Karen. Previous reports have shown that the prevalence of Mi^a^ antigen among Thai people varies by geographic region: frequencies of 4.7%, 10.2%, 17.97%, and 22.3% were found in southern, central, northeastern, and northern Thais, respectively (Intharanut et al., 2017; Romphruk et al., 2019; Khosidworachet et al., 2022). Overall, the data revealed that the distribution of blood group antigens varies significantly depending on ethnicity and geographic location.

At the allele level, across all studied populations with and without a previous history of malaria infection, we found GYP*Mur was the most common allele, followed by GYP*Thai. In contrast, we did not find the GYP*HF, GYP*Bun, GYP*Hop, or GYP*Kip alleles, consistent with previous studies (Nathalang et al., 2024). Therefore, we confirmed that GYP*Mur is the most frequent variant in the Thailand-Myanmar border area, which is similar to those previously reported from several regions in Southeast Asia (Chandanayingyong & Bejrachandra, 1975; Poole et al., 1991; Broadberry & Lin, 1996; Huynh et al., 2003; Hsu et al., 2013; Jongruamklang et al., 2018; Romphruk et al., 2019; Hassan et al., 2023; Nathalang et al., 2024). Even though GYP*Bun has been reported to be seen frequently in Southeast Asia after GYP*Mur, the GYP*Bun they are referring to was designated as GYP*Thai in our study (KR363627.1) (Wei et al., 2016b; Wei et al., 2018; Jongruamklang et al., 2020; Nathalang et al., 2024). Conversely, we did not obtain any of the reference GYP*Bun (M60710.1). Therefore, the GYP*Thai allele (KR363627.1) is far more common than the reference GYP*Bun allele (M60710.1) in the Thailand-Myanmar border area. Although the GYP*Thai allele was not found in Karen individuals, we identified the GYP*Thai allele with frequencies of 1.9% and 0.3% in northern Thais and Burmese, respectively. It suggests that this allele varies among populations.

Alloantibodies to hybrid glycophorin and Mi^a^ antigens are much more frequently implicated in immediate and delayed HTRs and HDFN in Southeast Asian than Caucasian and African populations (Poole & Daniels, 2007; Heathcote, Carroll & Flower, 2011; Makroo et al., 2016). Recently, an alloantibody (anti-JENU) in a GYP*Mur/GYP*Mur homozygote individual was identified in a Thai thalassemia patient. The JENU antigen (MNS49) is a new high-incidence antigen on GPB. Individuals with homozygous for GP.Mur lack normal GPB and their RBCs are JENU-, rendering them at risk of alloimmunization when exposed to RBCs carrying normal GPB (JENU+) (Lopez et al., 2017). In addition, GP.Mur and GP.Bun (encoded by GYP*Thai) are both qualitatively and quantitatively altered s antigen. A previous report noted that s antigen on GP.Mur or GP.Bun failed to react with IgM monoclonal anti-s (P3BER) but still reacted with polyclonal anti-s. In their study, alloanti-s was identified in a S+s+ patient with the GYP*Mur/GYPB*S genotype (Jongruamklang et al., 2020). Therefore, the accurate identification technique of hybrid glycophorins and Mi^a^ antigen is crucial.

Mi^a^ typing is routinely done using monoclonal anti-Mi^a^ (Romphruk et al., 2019). Commercial antibodies to type hybrid glycophorins remain unavailable due to the complexity of the antigens. Therefore, it is challenging to identify these hybrid glycophorins using serological methods and results may be inconclusive. Thus, various molecular techniques have been applied to overcome these challenges (Shih et al., 2000; Palacajornsuk et al., 2007; Wei et al., 2016a; Wei et al., 2018; Schoeman et al., 2017). In this study, we applied PCR-SSP combined with Sanger sequencing and HRM for genotyping analysis to determine the zygosity of *GYP* hybrid variants. All results obtained using the HRM assay were completely in concordance with those obtained from PCR-SSP combined with Sanger sequencing. Nevertheless, PCR-SSP is time-consuming and requires gel electrophoresis. Sanger sequencing is needed to further identify the alleles accurately. On the other hand, the HRM genotyping assay is a rapid, sensitive, and closed-tube genotyping method that is able to identify the exact genotype of known hybrid glycophorins and is capable of detecting novel variants (Wei et al., 2018). For these reasons, we suggest that the HRM method is suitable for routine genotyping. Accordingly, our molecular-based techniques can be used to identify hybrid glycophorins and Mi^a^ antigen in patients and donors. This approach may help to provide compatible blood products for transfusion and to prevent alloimmunization.

## Conclusions

Our study highlights the prevalence of glycophorin *GYP(B-A-B)* hybrids along the Thailand-Myanmar border region, among northern Thais, Burmese, and Karen with and without a previous history of malaria infection. Northern Thais have a higher frequency than Burmese and Karen people. However, we did not observe any difference between individuals with and without a previous history of malaria infection. GYP*Mur was the most common allele, which could potentially lead to alloimmunization *via* blood transfusions, organ transplants, or feto-maternal routes in these populations. The use of our molecular blood group genotyping approach may help in blood group screening in transfusion medicine to prevent alloimmunization.

## Supplemental Information

10.7717/peerj.19589/supp-1 Supplemental Information 1 Raw data for Tables 1 and 2

## Additional Information and Declarations

### DNA Deposition

The following information was supplied regarding the deposition of DNA sequences: The sequences of exon 3 and parts of the adjacent intron regions of *GYPA* are available at GenBank: NG_007470.3. The sequences of pseudoexon 3 and parts of the adjacent intron regions of *GYPB* are available at GenBank: M60708.1. The sequences of exon 3 and parts of the adjacent intron regions of *GYP(B-A-B)* including *GYP*HF*, *GYP*Mur*, *GYP*Bun*, *GYP*Thai*, *GYP*Hop*, and *GYP*Kip* are available at GenBank: M81079.1, AF090739.1, M60710.1, KR363627.1, KR815995.1, and KF501485.2, respectively.

### Data Availability

The following information was supplied regarding data availability: The raw measurements are available in the Supplemental File.
